# The Influence of Antipsychotic Treatment on the Activity of Abzymes Targeting Myelin and Levels of Inflammation Markers in Patients with Schizophrenia

**DOI:** 10.3390/biomedicines11041179

**Published:** 2023-04-14

**Authors:** Daria A. Kamaeva, Daria V. Kazantseva, Anastasiia S. Boiko, Irina A. Mednova, Liudmila P. Smirnova, Elena G. Kornetova, Svetlana A. Ivanova

**Affiliations:** 1 Mental Health Research Institute, Tomsk National Research Medical Center of the Russian Academy of Sciences, Aleutskaya Str. 4, Tomsk 634014, Russia; 2 Department of Psychiatry, Addictology and Psychotherapy, Siberian State Medical University, Moskovsky Trakt, 2, Tomsk 634050, Russia

**Keywords:** cytokine, schizophrenia, biomarker, catalytic IgG, abzymes, myelin, myelin basic protein, antibodies, humoral immunity

## Abstract

Catalytic antibodies, or abzymes, are capable of not only binding but also hydrolyzing various proteins. Previously, an increase in the level of myelin basic protein (MBP)-hydrolyzing activity of antibodies was shown in patients with a number of neurological and mental disorders, including schizophrenia. Furthermore, antipsychotic therapy is known to induce a change in cytokine levels in patients with schizophrenia, which affects regulation of the immune response and inflammatory status. This study investigated the influence of typical and atypical antipsychotics on catalytic antibody activity and the 10 major pro- and anti-inflammatory serum cytokine levels. The study included 40 patients with schizophrenia: 15 treated with first-generation antipsychotics and 25 treated with atypical antipsychotics for 6 weeks. It was found that treatment with atypical antipsychotics changed the levels of some pro-inflammatory cytokines. Antipsychotic therapy also caused a significant decrease in MBP-hydrolyzing activity in patients with schizophrenia (*p* = 0.0002), and associations of catalytic activity with interleukins were observed.

## 1. Introduction

Schizophrenia is a severe, multifactorial, heterogeneous mental disorder with an ambiguous etiopathogenesis, characterized by a progressive downhill course. Symptomatically, schizophrenia is expressed by the presence of positive (e.g., delusions and hallucinations), negative (e.g., anhedonia and social withdrawal), and affective symptoms, as well as cognitive deficits. According to the data obtained from positron emission tomography, in vitro and in vivo animal modeling, and post-mortem brain tissue studies, aberrant glutamate–dopamine synaptic and intracellular signaling interactions are central to the pathogenesis of schizophrenia [1,2]. Molecular abnormalities in the nervous [1], immune [3], and endocrine systems [4,5], and metabolic processes [6,7] also play an important role in the pathophysiology of the disease. Accumulating evidence also indicates that immunoinflammation may be involved in the pathogenesis of schizophrenia. Pro-inflammatory cytokine levels have been found to be elevated in the blood and cerebrospinal fluid of patients with schizophrenia [8]. The first episode of schizophrenia and the acute psychotic episode are accompanied by an increase in inflammatory parameters, including the levels of pro-inflammatory cytokines in the peripheral blood [9,10]. Neuroimaging studies have shown the activation of microglia, followed by the generation of inflammatory mediators. The impact of these modulators on neighboring neuronal and astrocytic cells makes a significant contribution to the homeostatic regulation of brain tissue [8,11,12]. Electron microscopic examination revealed local destruction of myelin sheaths and atrophy of axons in the prefrontal cortex, caudate nucleus, and hippocampus in schizophrenia patients [13,14]. Decreased density of white matter oligodendrocytes in the frontal part of the cortex [15] and hypomyelination of white and gray matter have also been revealed in schizophrenia [16]. Myelin volume loss and microglial activation, which have been clearly shown in neuroimaging studies, match the assumption of a low-grade inflammation and neurotoxicity in patients with schizophrenia [17]. Inflammatory damage [9] and oxidative stress [18], both of which occur in schizophrenia, may contribute to damage to cell membranes and induce the formation of autoantibodies. The immune system abnormalities in schizophrenia include changes in adaptive immunity and autoantibody production. In schizophrenia, a wide range of antinuclear antibodies, antineuronal antibodies [19], and antibodies to myelin are found [20]. Research by Kliushnik, T. et al. indicated that antibodies to MBP may be related to the diagnosis of schizophrenia [21,22], and associated with the severity of clinical symptoms [23]. The different dynamics of immune markers, including autoantibodies to MBP, correspond to different features of clinical remission after first-episode psychosis in young patients [24]. Among antigen-binding antibodies there are antibodies that can catalytically modify the antigen. Such antibodies have been identified in various mental disorders [25,26]. In patients with schizophrenia, catalytic antibodies (abzymes) hydrolyzing DNA [27], RNA, and miRNA [28], histones [29], and myelin basic protein (MBP) [20,30] have been previously found. Natural abzymes targeting proteins of the nervous system and myelin are an indicator of destructive processes, and they could potentially link the immune system and the extent of myelin damage [30].

The study of predictors of catalytic antibodies’ occurrence at the level of defects in hematopoiesis in autoimmune MRL-lpr/lpr mice showed specific reorganization of the immune system. This reorganization led to a change in the differentiation profile of hematopoietic stem cells in the bone marrow, with the production of DNA-, ATP-, and polysaccharide-hydrolyzing abzymes [31,32,33,34]. Genetic studies, on the other hand, indicated that the catalytic activity of immunoglobulins is germline-encoded, thus showing that catalysis is an innate function of an antibody, formed during phylogenetic evolution [35]. This hypothesis is supported by the presence of catalytic antibodies in healthy people [36] and catalytic antibodies against pathogenic antigens with a protective potential [37,38]. However, it is still unknown why, in a number of conditions, catalytic antibodies targeting self-antigens become more active. The catalytic activity of antibodies is a dynamic parameter which can be detected before the onset of clinical symptoms of disease in some cases [37], and which significantly decrease upon recovery or remission [20]. Thus, the question of the regulation of the catalytic activity of antibodies remains poorly understood. It is known that many of the conditions in which proteolytic antibodies have been detected are accompanied by immunological deregulation, affecting changes in the cytokine profile. Cytokines are the main signaling molecules of the immune system that determine the differentiation of T and B lymphocytes, the intensity of inflammation, and autoimmune reactions [8,12,39,40].

As mentioned above, a change in the cytokine profile is characteristic of schizophrenia and may change with antipsychotic therapy. Antipsychotics targeting neurotransmitter systems are the main treatment for schizophrenia. These medicines are principally categorized into two classes: first-generation antipsychotics (FGA), or typical antipsychotics, and second-generation antipsychotics (SGA), or atypical antipsychotics. Haloperidol is the most widely known FGA. The main representatives of SGAs include clozapine, risperidone, olanzapine, quetiapine, and others. SGAs are predominantly antagonists of 5-HT2A serotonergic and D2 dopamine receptors and partial agonists of 5-HT1A receptors, while the main mechanism of action of FGAs is associated with inhibition of D2 receptors [41,42]. Based on the receptor profile, the most common adverse events for FGAs are extrapyramidal symptoms, and for SGAs, are metabolic disorders [43,44,45]. Antipsychotic drugs are used for the long-term treatment of schizophrenia and have various effects on redox balance and inflammation. The effects of FGAs and SGAs on redox balance [18] and inflammation [44] vary significantly. Some research indicates that long-term treatment and high doses of first-generation antipsychotics reduce antioxidative defenses [46] and may cause oxidative stress [47], while atypical antipsychotics can affect the redox balance differently [48]. There is evidence of the anti-inflammatory effects of antipsychotics [42,49]. In first-episode patients, antipsychotic therapy decreased pro-inflammatory (such as IL-1β, IL-6, IFN-γ, TNF-α) and anti-inflammatory (such as IL-4, IL-10) cytokine concentrations, according to a meta-analysis [50]. Changes have been recorded in several additional candidate biomarkers (including S100B, prolactin, IL-2, insulin, leptin, IL-1 RA (receptor antagonist), IL-8, and IL-2 RA) [49]. Recent studies of antipsychotic influence on the myelination and development of the oligodendroglial lineage and their underlying molecular mechanisms suggest that the antipsychotics induce an improvement in myelin/oligodendrocyte-related dysfunction, which may contribute, at least in part, to their therapeutic effect on schizophrenia [51].

Previously, we demonstrated the presence of antibodies to myelin basic protein endowed with proteolytic properties in patients with schizophrenia [30], and the association of this activity with the clinical features of the disease [20]. In addition, we previously assessed the effect of SGAs on the serum concentration of cytokines, as well as the cytokines involved in metabolic syndrome development in schizophrenia [12,39].

The present study aimed to investigate the influence of typical and atypical antipsychotics on the activity of catalytic antibodies and the level of major pro- and anti-inflammatory cytokines.

## 2. Materials and Methods

### 2.1. Patients and Biological Material

A total of 40 schizophrenia patients aged 18 to 55 were included in the study after providing signed informed consent. The diagnosis was established according to the International Statistical Classification of Diseases and Related Health Problems, 10th Revision (ICD-10: F20). Participants with evidence of acute or chronic infectious, inflammatory, and autoimmune disorders, as well as individuals who used drugs, abused alcohol, or took medications that could affect immunological parameters, were excluded from the study. The observation period was six weeks, during which the patients were in the hospital. The level of severity of schizophrenia symptoms was evaluated using the Positive and Negative Syndrome Scale (PANSS) and the Clinical Global Impression scale (CGI) before and after the treatment.

Blood samples were collected from the cubital vein of all subjects after overnight fasting in the first days of hospitalization and after six weeks of treatment. Serum separation was performed by centrifugation at 2000× *g* for 20 min at 4 °C.

### 2.2. Quantitative Multiplexed Cytokine Analysis

Quantitative analyses of 10 serum cytokines (IL-1α, IL-1β, IL-1RA, IL-2, IL-4, IL-6, IL-10, IFN-y, TNF-α, IL-17A) were performed using xMAP technology with the multi-analyte panel HCYTMAG-60K-PX41 by MILLIPLEX MAP (Merck, Darmstadt, Germany) on the multiplex analyzers MAGPIX and Luminex 200 (Luminex, Austin, TX, USA), based at the Core Facility “Medical Genomics” Tomsk National Research Medical Center. Concentrations were measured in pg/mL, followed by data export to the xPONENT software (Luminex, Austin, TX, USA), and then export to the software Milliplex Analyst (Merck, Darmstadt, Germany).

### 2.3. Purification of Serum IgGs

Affinity chromatography on a column with Protein G-Sepharose using an ÄKTA pure chromatography system (GE Healthcare Bio-Sciences, Danderyd, Sweden), followed by gel filtration on a Superdex-200 HR 10/30 column, was used to isolate serum polyclonal IgG. Electrophoretic analysis in SDS-polyacrylamide gel with a gradient of 4–18% was used to confirm the homogeneity of the IgG.

### 2.4. Western Blotting of purified IgG

Purified serum IgGs from individual samples were separated and transferred to PVDF membranes using electroblotting in tris-glycine transfer buffer. The blot was then pre-incubated for 60 min with a blocking buffer and washed with phosphate-buffered saline (PBS) containing 0.05% Tween, before incubation with primary anti-human IgG antibodies at a 1:2000 dilution (Sigma Aldrich, St. Louis, MO, USA) at 4 °C. Following further washing with PBS-Tween, the blot was incubated with secondary antibodies for 60 min. The staining was performed with the Opti-4CN Substrate Kit. The visualization was performed on the iBright Imaging Systems FL1500 at the core facility of Medical Genomics (Tomsk Scientific Research Center).

### 2.5. Proteolytic Activity Assay

The proteolytic activity of the IgG was evaluated based on the extent of hydrolysis of the myelin basic protein in the presence of purified antibodies in vitro. The human brain myelin basic protein was provided by the Department of Biotechnology, Research Center of Molecular Diagnostics and Therapy in Moscow, and the hydrolysis was performed in the presence of purified antibodies. The reaction mixture (10 µL) contained 1 mg/mL MBP, 20 mM Tris-HCl (pH 7.5), and 0.2 mg/mL IgG. The mixture was incubated at 37 °C for 20 h. The products of protein cleavage were separated in 12% SDS-PAAG followed by Coomassie R250 staining. The color intensity of the MBP samples incubated in the absence of IgG was used to correct the values of IgG-induced hydrolysis. Gels were visualized using the iBright Imaging Systems FL1500 gel documentation system (Thermo Scientific, Waltham, MA, USA) on the basis of the Core Facility “Medical Genomics” (Tomsk NMRC). Quantitative evaluation of proteins was estimated using the iBright Analysis Software.

### 2.6. Statistical Analysis

Statistical analysis of the data was performed using Statistica 12.0 software for Windows. In our study, most of the data were not normally distributed according to the Shapiro–Wilk test for normality. Therefore, non-parametric tests, such as the Mann–Whitney U test for pairwise comparisons, the Wilcoxon’s test for dependent groups, and Spearman’s criteria for correlation analysis, were chosen for statistical analysis. Correlation analysis was performed by means of Spearman’s criteria. Data with *p*-values of less than 0.05 were considered statistically significant.

## 3. Results

### 3.1. Baseline Characteristics of Study Participants

The demographics and disease characteristics, as well as the antipsychotic therapy of the study population, are summarized in Table 1. A total of 40 patients with schizophrenia who received FGA and SGA were included in the study. Among patients in the FGA group, 60% were taking haloperidol, 20% were taking chlorprothixene, and 20% were taking zuclopenthixol. Most patients in the SGA group were taking risperidone (64%), quetiapine (24%), or olanzapine (12%) as their baseline antipsychotic therapy. Patients in groups with different drug therapies were comparable in terms of the main demographics and disease characteristics. Baseline disease severity, as assessed by CGI, was significantly higher in patients subsequently treated with FGAs (*p* = 0.0165) than in those subsequently treated with SGAs.

According to the CGI-Improvement score after medication, the patient’s overall clinical condition in all groups was classified as “much improved” [52]. For all patients, the course of therapy led to a decrease in PANSS scores (Table 2). Among patients treated with FGAs, 12 (80%) responded to therapy. Among patients treated with SGAs, 19 (76%) responded to therapy. Improvement was defined as at least a 20% reduction of the PANSS total score [53].

### 3.2. MBP-Hydrolyzing Activity of IgG Depending on the Antipsychotic Therapy

**Figure 1:**
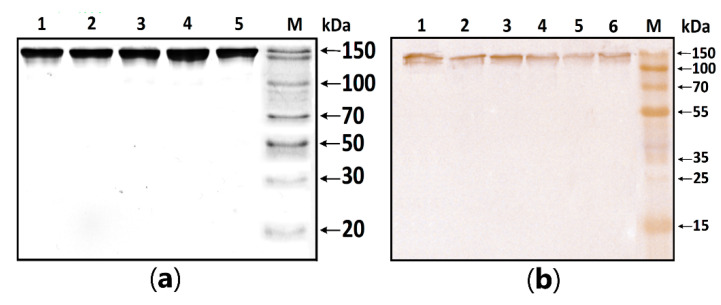
**Figure 1**. (**a**) SDS-PAGE analysis of a homogeneous IgG preparation in 12.5% gel followed by silver staining. Lane 1—intact IgG preparation; lane 2—IgG preparation incubated with 40 mM DTT at 100 °C (conditions for the complete reduction of disulfide bonds); lane M—protein molecular weight markers. (**b**) Western blotting of purified IgG. Lines 1–6: Immunostaining of purified polyclonal IgG with anti-human IgG antibody; M—protein molecular weight markers.

MBP-hydrolyzing activity was determined in serum IgG isolated according to the standard protocol published earlier [1,2,3]. In this work, the homogeneity of purified antibodies was assessed using electrophoresis in PAAG, followed by transfers to a PVDF membrane and staining with primary antibodies against human IgG (Figure 1). The test results showed the absence of co-isolated proteins in all identified bands, under both native and denaturing conditions, stained with antibodies against human IgG.

**Figure 2:**
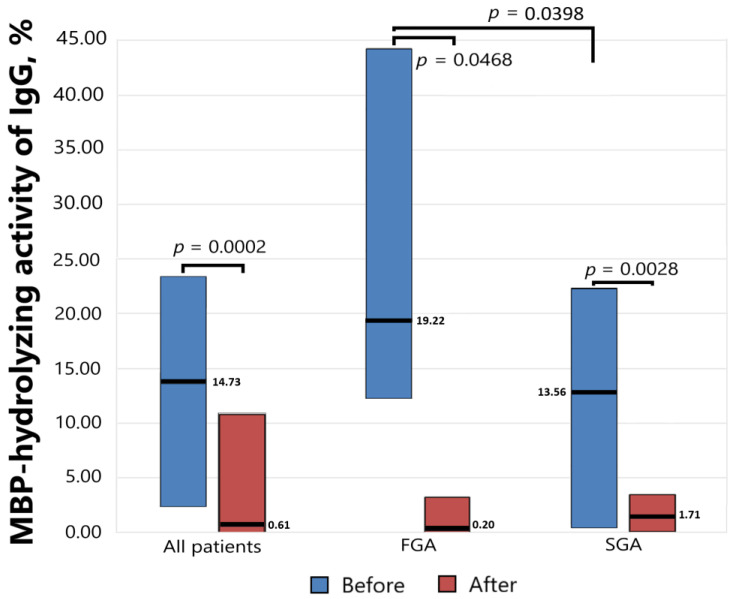
The MBP-hydrolyzing activity of IgGs from the sera of patients with schizophrenia treated with FGAs and SGAs for six weeks.

Antibody-dependent MBP-hydrolyzing activity was determined before starting antipsychotic therapy and six weeks after, and the results presented in Figure 2 show a significant decrease in the level of proteolytic activity as a result of drug therapy both for the general group (*p* = 0.0002) and for the subgroups of patients receiving FGAs (*p* = 0.0468) and SGAs (*p* = 0.0028). At the same time, the initial level of activity significantly differed (*p* = 0.0398) in the subgroups receiving FGAs and SGAs.

The level of MBP-hydrolyzing activity of IgGs was normalized to standard conditions (1 mg/mL MBP proteins; 0.2 mg/mL IgGs; 20 h of incubation at 37 °C). The complete hydrolysis of the MBP as substrates was taken as 100%. The significance of the differences (p) was calculated using the Mann–Whitney U test. MBP—myelin basic protein. FGA—first-generation antipsychotics. SGA—second-generation antipsychotics.

### 3.3. Cytokine Levels Depending on the Antipsychotic Therapy

In our study, treatment with typical and atypical antipsychotics affected the level of serum cytokines differently. In the group of patients treated with FGAs, the median values of anti-inflammatory cytokines IL-1RA, IL-4, and IL-10 increased, while a number of pro-inflammatory cytokines, including IL-1β and IL-17A, decreased, but without statistical significance (Table 3). At the same time, the levels of a number of pro-inflammatory cytokines increased, including IL-2, IFN-y, IL-6, and TNF-α.

**Figure 3:**
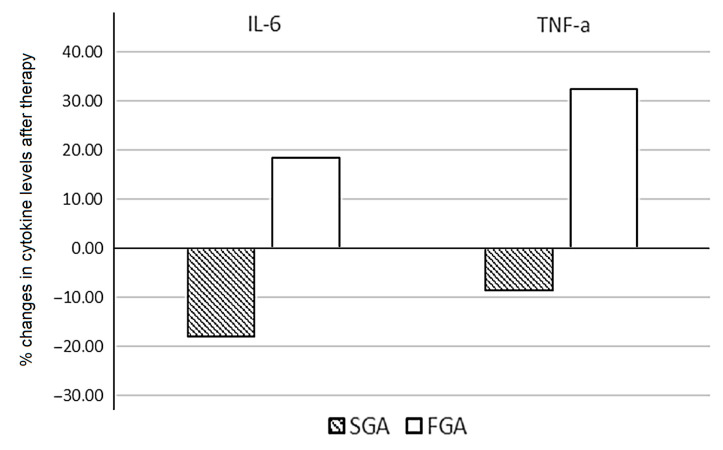
Percentage change in the cytokine levels after six weeks of FGA and SGA therapy in patients with schizophrenia.

Patients treated with SGAs demonstrated a significant decrease in levels of IL-6 (*p* = 0.036). The median values of IL-2, IFN-y, and TNF-α were reduced after therapy, without statistical significance. When assessing the percentage of changes in the cytokine levels between patients treated with FGAs and SGAs, statistically significant opposite changes in the levels of IL-6 (*p* = 0.033) and TNF-α (*p* = 0.036) were found (Figure 3).

We calculated the individual percentage changes in the indicator for each patient separately using the formula ((V2 − V1)/V1) × 100, where V1 is the initial value before therapy, and V2 is the final value after therapy. The data in the figure are presented as the median of the changes in the indicators. The figure shows only the statistically significant differences according to the Mann–Whitney U test.

### 3.4. Correlation Analysis of Changes in the Level of Cytokines and MBP-Hydrolyzing Activity during Antipsychotic Therapy

The correlation analysis did not reveal significant correlations in the subgroup of patients treated with FGAs. Results of correlations between changes in the level of cytokines and MBP-hydrolyzing activity during SGA therapy are presented in Table 4.

In the general group of patients, we observed a negative correlation between the initial level of MBP-hydrolyzing activity and IL-2 (r = −0.3313, *p* < 0.05). According to the correlation analysis, the initial level of MBP-hydrolyzing activity was negatively correlated with IL-2 (r = −0.4759, *p* < 0.05) and IFNy (r = −0.4558, *p* < 0.05) in patients treated with SGAs. A negative correlation of moderate strength (r = −0.390240, *p* < 0.05) was also found between baseline activity levels and CGI scores.

The level of MBP-hydrolyzing activity after SGA treatment was negatively correlated (r = −0.474074, *p* < 0.05) with chlorpromazine equivalents.

The percentage change in MBP-hydrolyzing activity in the SGA-treated group was positively correlated (r = 0.479554, *p* > 0.05) with the percentage reduction in IL-17A.

## 4. Discussion

In this work, for the first time, we studied the level of MBP-hydrolyzing activity of serum abzymes over the course of therapy and compared this parameter with the levels of cytokines. To assess the MBP-hydrolyzing activity, we used homogeneous serum IgG purified by affinity chromatography. Immunostaining with anti-IgG (human) confirmed that the obtained proteins were IgG. We found differences in the initial level of MBP-hydrolyzing activity in patients who subsequently received typical and atypical antipsychotics. These groups of patients differed in severity according to the CGI scale. In the group of patients subsequently treated with FGAs, CGI scores (*p* = 0.0165) and the level of MBP-hydrolyzing activity of serum IgG (*p* = 0.0398) were higher than in patients subsequently treated with SGAs. These differences were probably related to the severity of the disease, which was also indirectly confirmed by us in previous studies demonstrating the highest values of the activity of MBP-hydrolyzing antibodies with negative symptoms [30], during exacerbation of the disease, and in patients with continuous schizophrenia [20]. This conclusion was also confirmed by the presence of a positive correlation between the level of MBP-hydrolyzing activity at the beginning of treatment and CGI scores (r = 0.390240, *p* < 0.05).

Therapy with both typical and atypical neuroleptics led to a significant decrease in the level of MBP hydrolysis (*p* = 0.0002); however, the decrease in proteolytic activity was accompanied by various changes in the level of cytokines in the FGA- and SGA-treated groups. In contrast, the study of MBP-binding antibodies by Kliushnik et al. did not reveal any statistically significant differences in the MBP antibody levels before and after the treatment, although the dynamics of their changes were closely related to the therapy outcome, decreasing in responders and increasing in non-responders [23]. We have previously shown that the MBP-binding polyclonal serum IgG of schizophrenic patient isolated with affinity chromatography on MBP-Sepharose can be divided into several fractions with different affinities to MBP, but only one of those fractions was endowed with MBP-hydrolyzing activity (unpublished data). This proves that only some of the serum anti-MBP antibodies are MBP-hydrolyzing abzymes. Thus, the catalytic activity of antibodies can change regardless of the total pool of antigen-specific antibodies.

Patients treated with second-generation atypical antipsychotics showed a significant decrease in IL-6 levels (*p* = 0.036) as a result of therapy, as well as a relative decrease in TNF-α levels. In patients treated with FGAs, levels of these pro-inflammatory cytokines increased. In the group of patients treated with FGAs, the increase in cytokine levels before and after therapy had a statistically significant trend for IL-6 (*p* = 0.109) and TNF-α (*p* = 0.168). The percentage changes in the cytokine levels of IL-6 (*p* = 0.033) and TNF-α (*p* = 0.036) demonstrated significant differences between subgroups treated with different types of antipsychotics. These results were consistent with the in vivo and in vitro evidence for different effects of typical and atypical antipsychotics on the cytokine profile [43]. Larissa Daniele Bobermin, in an in vitro study of the effects of typical and atypical antipsychotics on astroglial cells, showed that an SGA (risperidone) was an anti-inflammatory agent, reducing the release of TNF-α, IL-1 β, and IL-6. However, haloperidol induced a pro-inflammatory response in astroglial cells, increasing the extracellular levels of TNF-α [54]. Atypical neuroleptics, such as risperidone, reduced serum IL-6 in schizophrenia patients [55], along with reducing IL-10 and TNF-α in first episode patients [56], which also coincided with the results of our study. Baumeister et al. (2016) demonstrated that FGAs (haloperidol and chlorpromazine) have been associated with inconsistent effects on IFN-γ, IL-4 IL-2, IL-10, and TNF-α levels, without a significant increase in IL-6, while SGAs have been associated with decreased concentrations of IL-6, IFN-γ, and TNF-α [57].

IL-6 functions include promotion of differentiation and production of antibodies by B cells, alteration of synaptic plasticity, and regulation of neurodevelopment and various behavioral effects related to eating, sleeping, and stress [40,58]. Meta-analysis results showed that in patients with schizophrenia before treatment, the serum IL-6 level was higher than in healthy individuals and in patients after antipsychotic treatment [58]. This interleukin stimulates acute phase responses, hematopoiesis, and immune responses in infections or tissue damage, but prolonged elevation promotes chronic inflammation and autoimmune responses [40].

The majority of studies have shown increased levels of TNF-α in the first episode of schizophrenia and during its exacerbations [12]. TNF-α has been shown to be involved in neuro-immune regulation, including the development and differentiation of glia and maintenance of normal brain morphology [59]. Additionally, the TNF-α and TNF-α-related signaling pathways may be crucial in the pathophysiology of schizophrenia [60]. TNF-α levels were significantly positively associated with PANSS negative symptoms in first-episode drug-naïve patients with schizophrenia, and were negatively correlated with the general psychopathology subscales and PANSS total scores in chronic patients with schizophrenia [60]. Furthermore, there was a positive correlation between serum TNF-α levels and cognitive function in schizophrenia patients [61]. The inappropriate or excessive activation of TNF-α signaling is associated with chronic inflammation, and it can eventually lead to the development of pathological conditions such as autoimmune diseases [62].

In our work, for the first time, we revealed the relationship between the MBP-hydrolyzing IgG activity level and the level of serum cytokines in schizophrenia patients. The initial level of MBP-hydrolyzing activity was negatively correlated with the level of IL-2 for the general group of patients, and with IL-2 (r = −0.4759, *p* < 0.05) and IFNy (r = −0.4558, *p* < 0.05) in patients treated with SGAs. The change of IL-2 levels in patients with schizophrenia was found to be an increase in patients with schizophrenia in some research [46], while others found a decrease [63,64]. During the acute period IL-2 changes significantly, while it tends to stabilize in patients in a stable stage [65]. IL-2 is a cytokine necessary for the survival and proliferation of regulatory T cells expressing Foxp3+. A reduced level of IL-2 can cause instability in T regulatory cells and result in the release of cytokines, which can worsen autoimmune conditions [39,66]. The results of the study of IFN-γ levels in schizophrenia are controversial; several studies have found no significant change in IFN-γ levels, whereas others show either an elevation or decrease in IFN-γ levels in schizophrenia patients [12]. In the context of the Th1/Th2 immune response, the negative correlation of MBP-hydrolyzing activity with IFN-γ levels may reflect a Th1/Th2 imbalance, with an increased Th2 response (or humoral immunity) occurring in schizophrenia [67].

The level of MBP-hydrolyzing activity after treatment (r = 0.390240, *p* < 0.05), as well as the percentage decrease in activity (r = 0.559091, *p* < 0.05), were positively correlated with chlorpromazine equivalents in the subgroup of patients receiving SGA. In our study, this may reflect a dose-dependent nature of the decrease in the activity of proteolytic abzymes during SGA therapy, suggesting an anti-inflammatory effect of antipsychotics working to depress abzymes.

Summarizing the data obtained on the inverse correlation of MBP-hydrolyzing activity with IL-2 and IFN levels, a decrease in the level of IL-6 and the level of MBP-hydrolyzing activity after SGA therapy, and the positive correlation (r = 0.479554, *p* > 0.05) between the percentage decrease in MBP-hydrolyzing activity and a percentage reduction in IL-17A after SGA, indirectly reflect the relationship between the cytokine network that regulates the Th17/Treg balance and the MBP-hydrolyzing activity of IgG. Previous studies have reported that IL-6 may regulate the Th17/Treg balance via depressing the differentiation of Treg cells [68]. IL-6 downregulation or overproduction can alter the balance between Th17 and Treg cells. Thus, IL-6 may contribute to the polarization of T helpers in Th17 lymphocytes producing IL-17 [10].

TNF is a bridging cytokine between innate and adaptive immunity in inflammation, exerting pleiotropic effects on regulatory T cells. At a highly pro-inflammatory area, TNF produced by activated monocytes depresses the suppressive effects of Treg cells and contributes to chronic inflammation [69]. Along with the suppressor function of Foxp3+ regulatory T lymphocytes to other immune effector cells, it may also produce pro-inflammatory cytokines, such as IL-17A, in inflammatory conditions and corresponding cytokine environments [70,71]

Our study has a number of limitations. The lack of significant differences in the change of cytokine levels after therapy in the group of patients treated with FGAs can probably be explained by the small sample size. A more detailed evaluation of the relationship between the proteolytic activity of antibodies and the level of serum cytokines requires studies with a larger sample size. Data on the psychotherapy protocols were not available in this study, which limits the description of the patient group. We also did not take into account the impact of metabolic syndrome, smoking, and caffeine in our data analysis. The group of patients treated with FGAs included three people with metabolic syndrome. As has been shown previously, metabolic syndrome can significantly affect the level of cytokines, demonstrating multidirectional changes in groups of patients with and without metabolic syndrome who receive the same therapy [44]. The other limitation was the inability to fully evaluate long-term antipsychotic therapy before current hospitalization due to a lack of relevant information. In addition, the effect of antipsychotics on cytokine levels may depend on the duration of the disease. In first-episode drug-naïve patients with schizophrenia, antipsychotic treatment may decrease inflammatory cytokines, while prolonged use of antipsychotic medication can lead to the development of metabolic abnormalities and may increase inflammatory marker levels [43].

## 5. Conclusions

We found, for the first time, that the MBP-hydrolyzing activity of IgGs decreases during short-term antipsychotic therapy in patients with schizophrenia. The results of our work also suggest possible multidirectional correlations between the MBP-hydrolyzing activity of serum IgG and the level of serum cytokines. This highlights the need for further studies of the anti-inflammatory effects of antipsychotic drugs and the role of stabilization of immunological parameters in the clinical improvement of patients with schizophrenia.

## Figures and Tables

**Table 1:** Sociodemographic, clinical, and pharmacological characteristics of patients with schizophrenia, depending on antipsychotic treatment.

Indicators	All Patients (n = 40)	Patients Treated with FGAs (n = 15)	Patients Treated with SGAs (n = 25)	*p*-Value
Age, Me (Q1; Q3) years	35.00 (29.00; 39.50)	35.00 (31.00; 41.00)	35.00 (27.00; 39.00)	0.302
Gender (Male, n (%)/Female, n (%))	19 (47%)/21 (53%)	7 (45%)/8 (55%)	12 (48%)/13 (52%)	0.936
Schizophrenia onset age, Me [Q1; Q3] years	21.00 (19.00; 26.00)	23.50 (19.00; 25.00)	20.00 (20.00; 26.00)	0.849
Duration of disease, Me [Q1; Q3] years	10.50 (4.00; 16.00)	15.00 (7.00; 19.00)	9.00 (2.00; 15.00)	0.183
Total antipsychotic dose, CPZeq	300.00 (200.00; 393.75)	337.50 (200.00; 1 550.00)	300.00 (200.00; 355.00)	0.370
Antipsychotic therapy duration, years	3.50 (0.50; 10.00)	6.00 (1.00; 12.00)	2.50 (0.30; 9.00)	0.281
PANSS positive symptoms score	21.50 (16.00; 25.00)	23.00 (19.00; 26.00)	20.50 (15.50; 24.00)	0.261
PANSS negative symptoms score	25.50 (20.00; 29.00)	26.50 (19.00; 31.00)	24.50 (20.50; 28.00)	0.758
PANSS general psychopathology symptoms score	52.50 (43.00; 58.00)	51.00 (48.00; 57.00)	54.50 (41.50; 58.00)	0.965
PANSS total score	99.50 (86.00; 111.00)	99.50 (94.00; 112.00)	99.50 (82.50; 110.50)	0.659
CGI-Severity score	5.00 (4.00; 5.00)	5.00 (5.00; 6.00)	4.50 (4.00; 5.00)	0.017 *
CGI-Improvement score	2.00 (1.00; 2.00)	2.00 (2.00; 2.00)	2.00 (1.00; 2.00)	0.711

Note: Me (Q1; Q3)—median (lower quartile; upper quartile). PANSS—positive and negative syndrome scale. CGI—Clinical Global Impression Scale. CPZeq—Chlorpromazine equivalent. FGAs—first-generation antipsychotics. SGAs—second-generation antipsychotics. * *p* < 0.05—statistically significant difference. Comparisons between groups were performed using the chi-square test for gender and the Mann–Whitney U test for the other indicators.

**Table 2:** Clinical characteristics of patients with schizophrenia before and after therapy, depending on antipsychotic treatment.

	Patients Treated with FGAs (n = 15)			Patients Treated with SGAs (n = 25)		
Indicators	Before	After	*p*-Value	Before	After	*p*-Value
PANSS positive symptoms score	23.00 (19.00; 26.00)	13.5 (11.00; 15.00)	0.005 *	20.50 (15.50; 24.00)	11.50 (9.00; 17.00)	<0.001 *
PANSS negative symptoms score	26.50 (19.00; 31.00)	17.5 (16.00; 20.00)	0.005 *	24.50 (20.50; 28.00)	19.00 (13.50; 21.50)	<0.0001 *
PANSS general psychopathology symptoms score	51.00 (48.00; 57.00)	33 (28.00; 40.00)	0.005 *	54.50 (41.50; 58.00)	37.00 (24.50; 45.00)	<0.0001 *
PANSS total score	99.50 (94.00; 112.00)	64 (59.00; 77.00)	0.005 *	99.50 (82.50; 110.50)	70.00 (50.50; 82.50)	<0.0001 *

Note: Me (Q1; Q3)—median (lower quartile; upper quartile). PANSS—positive and negative syndrome scale. FGA—first-generation antipsychotics. SGA—second-generation antipsychotics. * *p* < 0.05—statistically significant difference. Comparisons between groups were performed using the Wilcoxon signed-rank test.

**Table 3:** Serum concentrations of the cytokines (pg/mL) in patients treated with FGAs and SGAs for six weeks, Me (Q1; Q3).

	Patients Treated with FGAs (n = 15)			Patients Treated with SGAs (n = 25)		
Indicators	Before	After	*p*-Value	Before	After	*p*-Value
IL-1α	51.19 (50.09; 54.67)	51.19 (47.66; 60.54)	0.515	54.53 (45.43; 60.75)	54.58 (45.01; 58.79)	0.658
IL-1β	2.84 (2.56; 3.10)	2.70 (2.33; 3.12)	0.333	2.49 (2.12; 2.98)	2.18 (1.73; 2.64)	0.158
IL-1RA	44.45 (36.21; 60.68)	49.72 (38.13; 72.45)	0.878	42.93 (33.27; 62.65)	43.53 (37.04; 62.89)	0.793
IL-2	4.83 (4.48; 5.63)	5.15 (4.20; 5.88)	0.441	4.69 (4.18; 5.40)	4.67 (4.34; 5.50)	0.525
IL-4	81.48 (70.37; 99.72)	85.33 (73.71;113.91)	0.959	76.69 (65.74; 95.20)	79.28 (61.82; 87.49)	0.778
IL-6	4.60 (4.00; 5.75)	5.86 (4.00; 6.31)	0.109	5.71 (4.43; 9.69)	4.77 (4.00; 6.58)	0.036 *
IL-10	7.39 (6.24; 8.61)	7.70 (6.21; 12.10)	0.575	7.93 (7.08; 9.05)	7.32 (6.39; 8.31)	0.248
IFN-y	10.59 (8.32; 12.49)	10.77 (9.93; 12.89)	0.888	11.31 (9.52; 13.13)	10.15 (9.34; 11.60)	0.286
TNF-α	17.04 (13.13; 29.88)	24.02 (17.51; 36.34)	0.168	15.42 (9.71; 25.48)	13.85 (10.48; 20.72)	0.573
IL-17A	5.48 (4.61; 7.28)	5.15 (4.61; 5.84)	0.779	4.61 (3.75; 6.47)	4.48 (3.65; 5.24)	0.778

Note: Me (Q1; Q3)—median (lower quartile; upper quartile). IFN—interferon; IL—interleukin; TNF—tumor necrosis factor. FGAs—first-generation antipsychotics. SGAs—second-generation antipsychotics. * *p* < 0.05 for the Wilcoxon signed-rank test.

**Table 4:** Spearman’s correlation analysis of the cytokine levels and MBP-hydrolyzing activity of IgG before and after SGA therapy.

	Before SGA Therapy	After SGA Therapy	Percentage Change after SGA Therapy ^#^
IL-1α	−0.193596	−0.075766	0.046111
IL-1β	−0.064283	−0.038396	0.161388
IL-1RA	−0.142747	0.041467	−0.431906
IL-2	−0.4759 *	0.025449	−0.207499
IL-4	−0.188308	0.030333	−0.010800
IL-6	−0.173181	−0.018930	−0.179833
IL-10	−0.106625	−0.344219	0.148435
IFN-y	−0.4558 *	−0.223136	0.101444
TNF-α	0.055127	−0.076624	0.132185
IL-17A	−0.068622	−0.194404	0.479554 *
total CPZeq		−0.474074 *	0.559091
	**MBP-hydrolyzing activity of IgG, %**		

Note: Data are presented as the correlation coefficient (r). MBP—myelin basic protein. CPZeq—Chlorpromazine equivalent. SGA—second-generation antipsychotics. ^#^ Percentage change of cytokine levels and percentage decrease of MBP-hydrolyzing activity of IgG after SGA therapy. * *p* < 0.05 for the Spearman’s correlation test. The results of correlation analysis are given without correction for multiple comparisons.

## Data Availability

The data presented in this study are available on request from the corresponding author.
